# Visualizing novel connections and genetic similarities across diseases using a network-medicine based approach

**DOI:** 10.1038/s41598-022-19244-y

**Published:** 2022-09-01

**Authors:** Brian Ferolito, Italo Faria do Valle, Hanna Gerlovin, Lauren Costa, Juan P. Casas, J. Michael Gaziano, David R. Gagnon, Edmon Begoli, Albert-László Barabási, Kelly Cho

**Affiliations:** 1 VA Boston Healthcare System, Massachusetts Veterans Epidemiology and Research Information Center, (MAVERIC), 150 S. Huntington Avenue, Boston, 02130 USA; 2 Center for Complex Network Research, Department of Physics, Northeastern University, Boston, 02115 USA; 3 School of Public Health, Department of Biostatistics, Boston University, Boston, 02215 USA; 4 Oak Ridge National Laboratory, Oak Ridge, 37830 USA; 5 Brigham and Women’s Hospital, Division of Aging, Department of Medicine, Harvard Medical School, Boston, 02115 USA

**Keywords:** Genetic variation, Computational biology and bioinformatics, Network topology, Genetic association study, Genome-wide association studies

## Abstract

Understanding the genetic relationships between human disorders could lead to better treatment and prevention strategies, especially for individuals with multiple comorbidities. A common resource for studying genetic-disease relationships is the GWAS Catalog, a large and well curated repository of SNP-trait associations from various studies and populations. Some of these populations are contained within mega-biobanks such as the Million Veteran Program (MVP), which has enabled the genetic classification of several diseases in a large well-characterized and heterogeneous population. Here we aim to provide a network of the genetic relationships among diseases and to demonstrate the utility of quantifying the extent to which a given resource such as MVP has contributed to the discovery of such relations. We use a network-based approach to evaluate shared variants among thousands of traits in the GWAS Catalog repository. Our results indicate many more novel disease relationships that did not exist in early studies and demonstrate that the network can reveal clusters of diseases mechanistically related. Finally, we show novel disease connections that emerge when MVP data is included, highlighting methodology that can be used to indicate the contributions of a given biobank.

## Introduction

Disease comorbidity, or the co-occurrence of diseases within a single individual, is a major clinical problem, posing challenges in prognosis and treatment, increasing health care costs, and reducing life expectancy^1,2^. Comorbidities suggest common mechanisms that underlie different diseases, which can be either genetic or environmental^3^. Recent network-medicine based approaches have systematically studied the relationships across hundreds of diseases, using either molecular or clinical data. For example, Goh et al.^4^ created a network in which diseases are connected if they are associated to the same gene or genetic variant, and Hidalgo et al.^5^ built a network that mapped all correlations observed in the medical records of millions of patients. These approaches have the power to reveal insights that are not apparent when diseases are studied in isolation, offering a holistic approach to investigate diseases and how they are related. In fact, network-medicine based approaches have highlighted groups of disorders connected to the same molecular and metabolic mechanisms^4,6–8^, comorbidities driven by age^9^, gender^9–11^, demographic factors^5^ or by the same environmental triggers^12^.

Recent advances in technology and computing power have allowed an exponential growth of data obtained by profiling thousands of patients. Large genomics initiatives across the world, such as the UK Biobank^13,14^; Kaiser Permanente Research Program on Genes, Environment, and Health^15^; China Kadoorie Biobank^16^; and others, have profiled millions of patients through Genome-Wide Association Studies (GWAS), increasing our ability to investigate and understand the molecular and genetic origins of diseases. The Million Veteran Program^17^ (MVP) is one of such initiatives, which covers 825,000 patients in the United States from diverse ancestry backgrounds. At the current state, over 35 MVP research projects^18^ cover a wide range of high priority research areas including cardiovascular disease, mental health, substance abuse, cardiometabolic disease, urogenital disorders, diseases of the nervous system, cancer, pharmacogenomics, metabolism, infectious disease, and pain.

Mega-biobank repositories, as MVP’s, contribute to the larger knowledgebase of genetic and disease mechanisms, and there is interest in being able to isolate the important and novel contributions of such initiatives to better target future efforts and research. Network-based approaches allow for the comparison of connected components that can also be further leveraged to focus causal inference towards genetic druggable targets, as well as, identifying pathways that are unique due to population stratification or genetic ancestries. We start by summarizing the current knowledge of genetic variants present in the GWAS Catalog, a curated public repository of genetic variant-phenotype associations from GWAS studies^19^. From the GWAS Catalog, we built a network where nodes represent single conditions and links represent shared genetic variants between a pair of diseases. We identified clusters of diseases based on the patterns of shared variants and compared the identified clusters with classical disease organization based on anatomical system. We apply the novelty-comparison method to discover novel disease relationships for conditions, due to MVP’s contribution, such as peripheral arterial disease, diabetes mellitus, and gout. Additionally, we show that these findings provide not only a high-level overview of our current understanding of genetic relationships among diseases, but also indicate new directions for further in-depth investigation, especially within particular ancestries, possibly offering new strategies for disease treatment and prevention.

## Results

### GWAS Catalog phenotypic network

**Figure 1:**
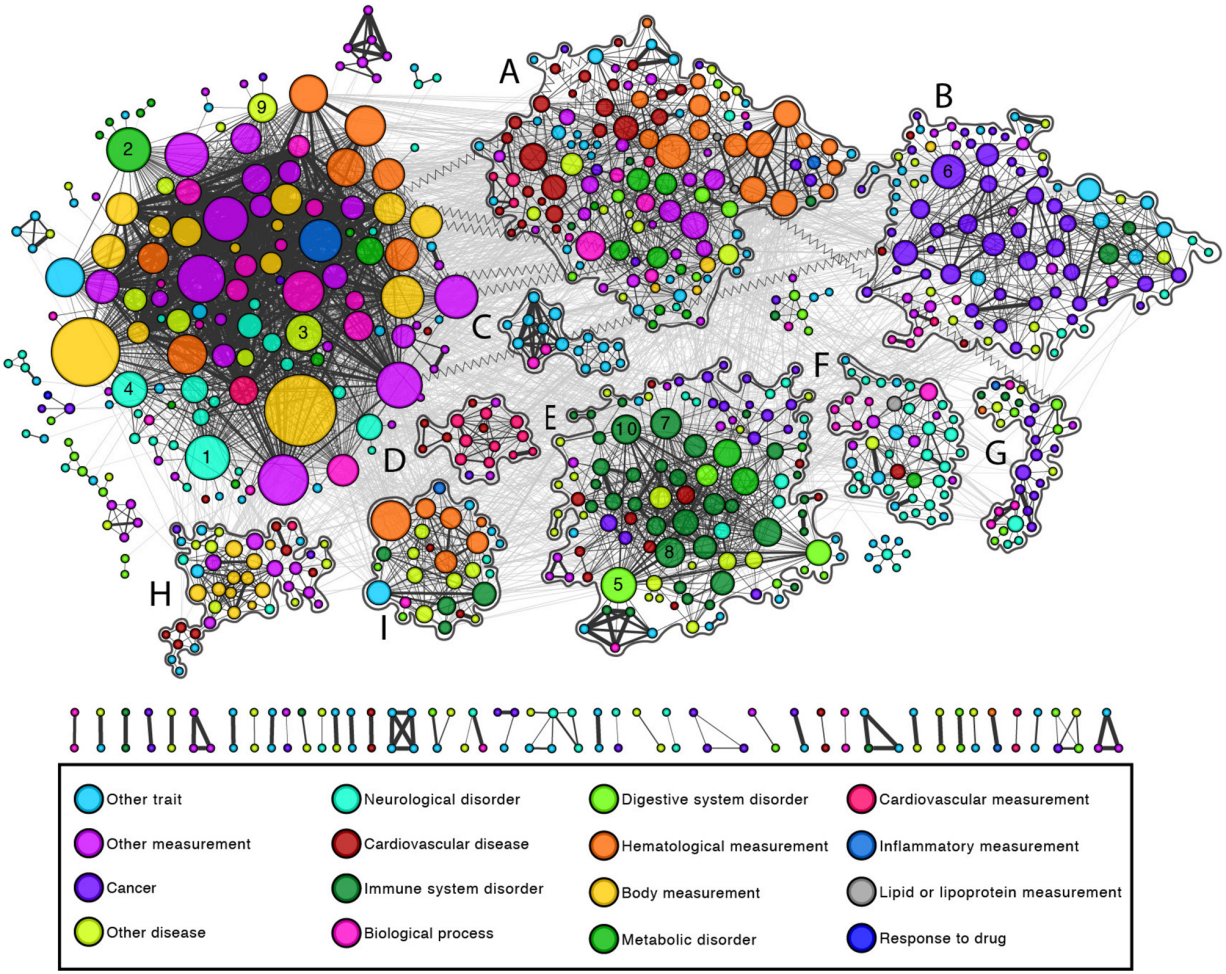
Phenotypic network assembled from GWAS catalog. Network in which nodes are traits that are connected with others to which they share genetic variants in the GWAS Catalog. The network communities detected are highlighted and labeled (**A**)–(**H**). Node colors represent disease categories and node size reflects connectivity in the network. The top high degree nodes are labeled 1–10 and their respective names are shown in Table 1. Only significant edges are shown (FDR < 0.05), the edge width indicates the overlap of variants between a pair of phenotypes (Jaccard Index), and lighter shade edges connect nodes in different communities.

We started by characterizing disease relationships arising from shared genetic variants among several diseases. To achieve this, we retrieved data from the GWAS Catalog, a curated public repository of variant-phenotype associations from eligible GWAS studies^19^. As of July 1st, 2020, the repository consisted of 3985 publications representing 113,841 genetic variants for 4298 unique traits. In this study, we focused only on 2764 disease-related traits from the full GWAS catalog, which included data from MVP as well as other sources. We eliminated many traits not directly associated with diseases from the analysis (See Methods). We then built a network in which nodes represent traits and links (or edges) connect traits that share variants. Each link contains a normalized measure of variant overlap between disease pairs (Jaccard Index), with its statistical significance being measured by the Fisher’s Exact Test followed by Benjamini–Hochberg multiple testing correction, and links with q > 0.05 are filtered from the network. The final network contains 810 traits and 4980 links (Fig. 1). Node information and edge list for the Phenotypic Network can be found in Supplementary Tables 1 and 2, respectively.

**Figure 2:**
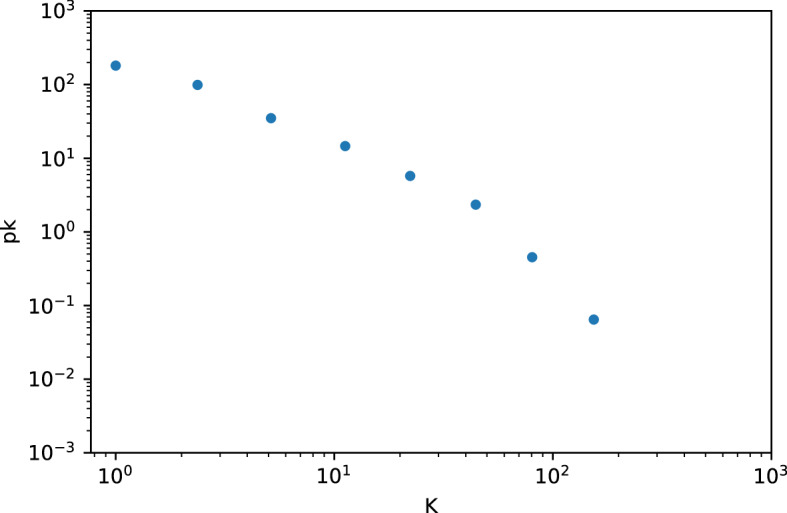
Degree distribution of phenotypic network. Log-binned degree distribution of the phenotypic network using a log–log scale. A power-law distribution which is a feature of scale-free networks. K represents the average degree of the bin where bin has size 2^n-1^. pK is obtained from the number of nodes found in the bin divided by the width of the bin.

**Figure 3:**
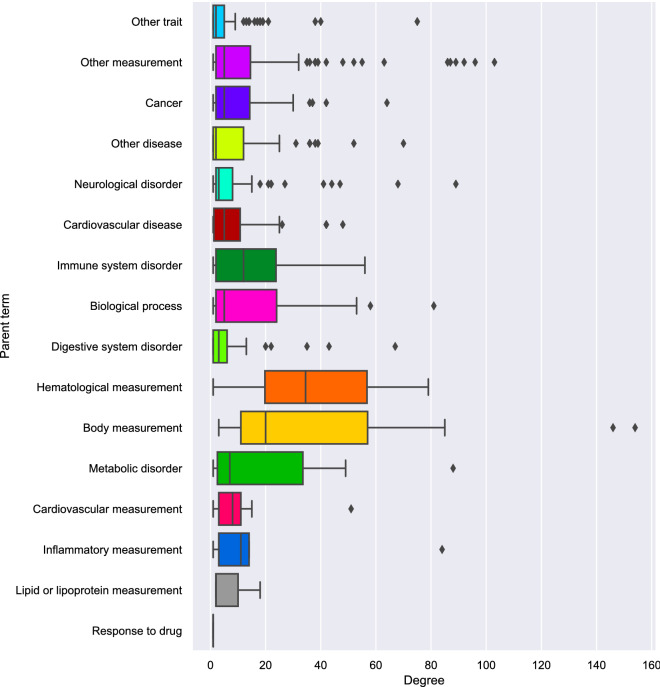
Degree distribution by trait category. Trait categories are defined by the EFO ontology system parent terms.

In the overall phenotypic network, the traits with highest connectivity (k) were body mass index (k = 154), body height (k = 146), and systolic blood pressure (k = 103) as these are common anthropometric measurements included in large number of analyses. Specifically, for diseases, the most connected were schizophrenia (k = 89), type II diabetes mellitus (k = 88), and asthma (k = 70) (Table 1). As commonly observed in biological networks, our phenotypic network has a power law degree distribution, resulting in a network with a few nodes connected to many others, while most nodes have only a few connections (Fig. 2). The trait categories with the highest degree nodes were hematological and body measurements (Fig. 3). The pairs of traits with the highest overlap of genetic variants were systolic and diastolic blood pressure (1535); adolescent idiopathic scoliosis and scoliosis (1368); and basophil and neutrophil count (1076). We observed a high correlation between disease connectivity and total number of variants (Pearson $\rho$= 0.866, *p* = 2.5 × 10^–245^) as well as disease connectivity and number of studies for the disease (Pearson $\rho$ = 0.672 and *p* = 1.45 × 10^–107^). These correlations with disease connectivity indicate that increased genetics data availability may make it more feasible to discover disease relationships not known before.

**Table 1:** High degree nodes of the phenotypic network.

Node	Degree	Centrality	Total variants	Responsible variants	Studies
Schizophrenia	89	0.141092	2497	1102	74
Type II diabetes mellitus	88	0.126487	1817	693	120
Asthma	70	0.119657	1617	870	66
Unipolar depression	68	0.119174	1763	954	64
Crohn's disease	67	0.090566	810	630	40
Breast carcinoma	64	0.097225	1046	225	66
Rheumatoid arthritis	56	0.058468	498	165	44
Ulcerative colitis	54	0.080451	692	595	27
Chronic obstructive pulmonary disease	52	0.1032	961	612	24
Psoriasis	52	0.068427	534	433	18

Table showing the top 10 most connected nodes, their corresponding eigenvector centrality, the total number of variants found for that trait, the number of those variants that are shared with other traits, and the number of unique papers reported for the traits in the database.

This can be demonstrated by comparing our results to previous disease networks. For example, Goh et al.^4^ mapped disease relationships using data from the Online Mendelian Inheritance in Man (OMIM) database. The authors report 7 diseases connected to schizophrenia and 11 connected to asthma, while our results report 89 and 70 connections, respectively.

Our results also highlight variants that connect the greatest number of disease pairs (Table 2). For example, the variant rs3184504 is shared between 641 disease pairs. This Single Nucleotide Polymorphism (SNP) is a missense variant found in the SH2B3 gene, which is a negative regulator of cytokine signaling, and an important component of the hematopoiesis pathway^20^. The diseases in our network that contain the most edges with this variant are type I diabetes mellitus, rheumatoid arthritis, multiple sclerosis, inflammatory bowel disease, colorectal cancer, and prostate carcinoma.

**Table 2:** Top variants found in the phenotypic network.

Variant	Chromosome	Edges	Gene
RS3184504	12	641	ATXN2, SH2B3
RS1260326	2	533	GCKR
RS12075	1	443	ACKR1, CADM3-AS1
RS516246	19	322	FUT2
RS8040868	15	311	CHRNA3
RS10830963	11	307	MTNR1B
RS2476601	1	278	AL137856.1, PTPN22
RS701428	22	276	LINC00896—RTN4R
RS3919627	3	276	AC092042.3, KRBOX1, AC099329.2, CYP8B1, ACKR2
RS700750	7	274	AC011294.1

Table showing the variants responsible for creating the greatest number of edges in the Phenotypic Network. Information includes the number of edges and the gene associated with that variant. The gene-variant relationships are acquired from the GWAS Catalog. For variants occurring in intergenic regions, both the upstream and downstream genes are shown.

### Disease clusters

The identification of groups of diseases that are mechanistically related can offer insights about disease comorbidity and lead to better strategies for disease treatment and prevention. Here, we leveraged the patterns of connections in the Phenotypic Network to reveal diseases that are closely related. We applied the community detection algorithm Louvain^21^, which seeks to find groups of nodes more connected among themselves than with the rest of the network. We highlight that this method considers only the pattern of connections in the network and does not take disease classification into account. The largest connected component of our network is comprised of 22 communities with the remaining 39 communities occurring in isolated nodes. We focus our discussion of the communities on disease-related traits, i.e. not considering all traits classified in the following categories: other measurement, biological process, body measurement, lipid or lipoprotein measurement, response to drug, and hematological measurement (see Methods). Our results are consistent with previous findings^4^ that clusters tend to aggregate diseases that share underlying mechanisms such as cancer, neurological, cardiovascular, and immune system disorders (Fig. 1).

**Figure 4:**
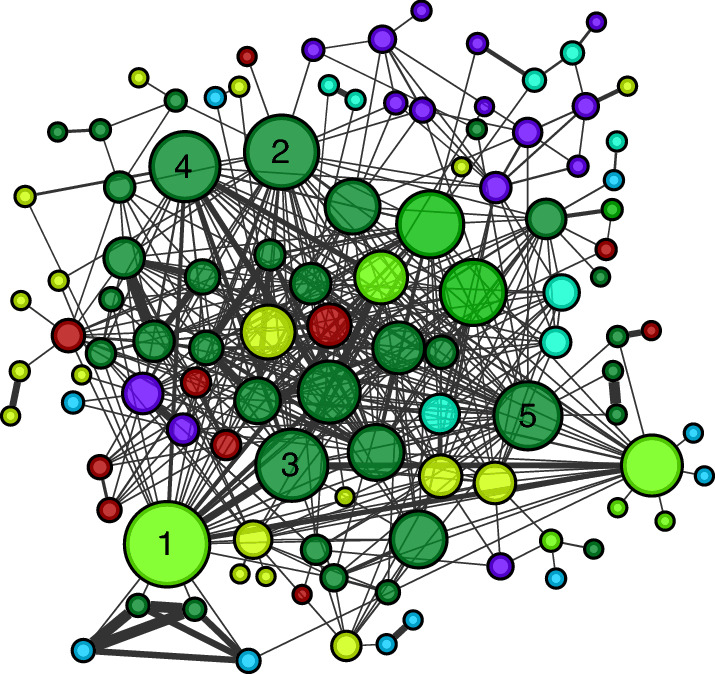
Network community ‘E’ characterized by immune-related disorders. Focused subgraph of community E from the Phenotypic Network. The most connected diseases in the community are Crohn’s disease (**1**), rheumatoid arthritis (**2**), ulcerative colitis (**3**), psoriasis (**4**), and lupus (**5**).

Community E, the community with the most disease-related traits (*n* = 105) is characterized by disorders of the immune system and the most connected diseases in the community are Crohn's disease, rheumatoid arthritis, ulcerative colitis, psoriasis, and lupus (Fig. 4). It also highlights conditions classically characterized in other disease groups (e.g., cancer, neurological disorders) that are known to be related to the immune system, for example, cancers associated with immune cells, such as B-cell or Hodgkin's lymphoma. Interestingly, COVID-19 is also in this community, connected only to Type I Diabetes by the common variant rs657152 in the ABO gene. Indeed, studies have reported relationship association of the ABO blood groups with type I diabetes^22,23^ and to different levels of susceptibility to SARS-COV-2 infection^24–26^. It is important to note that our data are limited to GWAS studies added to the GWAS catalog before June 30th, 2020, which were the early stages of the pandemic, and therefore more connections may be discovered with additional research.

**Figure 5:**
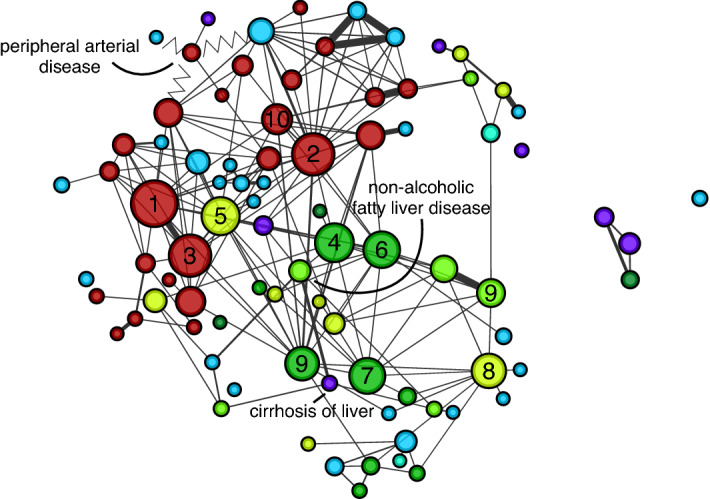
Network community ‘A’ characterized by vascular disorders. After removal of traits unrelated to diseases from the visualization, the most connected nodes in the community were coronary heart disease (**1**), stroke (**2**), coronary artery disease (**3**), metabolic syndrome (**4**), cardiovascular disease (**5**), hypertriglyceridemia (**6**), gout (**7**), chronic kidney disease (**8**), diabetes mellitus (**9**), and atrial fibrillation (**10**).

Community A, the second community with most disease-related traits nodes (*n* = 90) is characterized by diseases of the vascular system (Fig. 5). The most connected nodes in the community were coronary heart disease, stroke, coronary artery disease, metabolic syndrome, cardiovascular disease, hypertriglyceridemia, gout, chronic kidney disease, diabetes mellitus, and atrial fibrillation. Peripheral arterial disease (PAD), cirrhosis of liver, and non-alcoholic fatty liver disease are also in this community, and previous studies report association among these diseases^27,28^.

Community B, the third biggest community (*n* = 85) is characterized by several types of cancer, such as breast and ovarian serous carcinoma. This community also contains skin-related traits, such as vitiligo, sunburn, skin and hair pigmentation, and skin cancer. Retrospective studies in Taiwan and Korea have found increased risk of different types of cancer in patients with vitiligo^29,30^, and vitiligo-related genes have been linked to skin cancer^31^.

Finally, the network shows that Type II Diabetes is in the same community as several neurological disorders, such as Alzheimer's disease and schizophrenia. In fact, previous studies show that Type II Diabetes is linked to Alzheimer's disease and dementia^32–37^, and several anti-diabetic drugs can promote neuronal survival and lead to clinical improvement of cognition and memory^38^.

Altogether, these results demonstrate the intricate molecular relationships among diseases and how a network-based approach can help identify groups of diseases with shared underlying mechanisms. These communities might offer insights on specific comorbidity patterns observed in patients, as well as highlight genetic variants for future functional in-depth research.

### Novel disease relationships emerging from MVP findings

**Figure 6:**
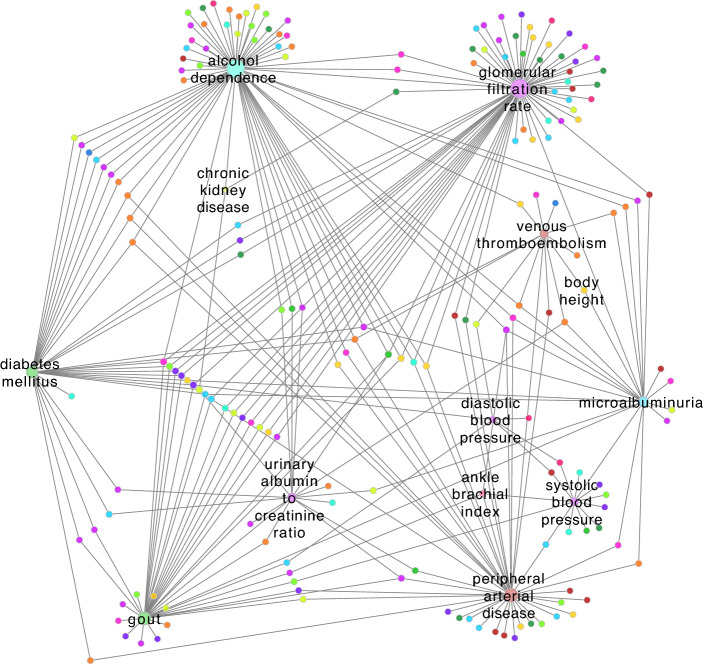
Disease connections that emerge from MVP data. Subgraph containing 196 traits and 297 edges that were formed only by the inclusion of genetic variant associations from the Million Veteran Program.

Large and representative cohorts allow for the discovery of new genetic variants associated with different conditions, especially amongst minority populations with diverse ancestries. In particular, the MVP cohort contains higher percentages of minority groups that are usually underrepresented in genetic studies^17,39^, which lead to the discovery of variants not observed in more homogeneous populations. For example, PAD had 167 variants reported in the GWAS Catalog from non-MVP sources, but an MVP study^40^ found 18 loci that were novel at the time of the publication. Out of these novel loci, four (rs2107595, rs505922, rs6025, rs7903146) were also observed for duodenal ulcers, glycosuria, large artery stroke, and ischemic stroke, revealing molecular links between diseases that were not observed before. Therefore, we sought to characterize the new relationships among diseases that emerge when genetic data from MVP studies obtained from the GWAS Catalog is integrated in the analysis. We analyzed the subnetwork formed only by edges exclusively created from MVP data, which contains 196 traits and 297 edges (Fig. 6).

The disease traits for which we identified the greatest number of novel disease relationships were, in descending order: glomerular filtration rate^41,42^, alcohol dependence^43^, peripheral arterial disease^40^, gout^44^, diabetes mellitus^42^, microalbuminuria^45^, urinary albumin to creatine ratio^45^, systolic blood pressure^46,47^, venous thromboembolism^40,48^, diastolic blood pressure^46,47^, and body height^49^ (Fig. 5). Glomerular filtration rate was the trait with the most novel edges, in which two MVP studies^41,42^ found 664 variants that created 19 new connections in the network. Traits evaluated by MVP studies that did not produce novel connections in the network were anxiety^50^, anxiety disorder^50^, bipolar I disorder^51^, schizophrenia^51^, ankle brachial index^40^, and panic disorder^50^. MVP publications found in the GWAS Catalog, the Phenotypic Network, and the MVP Novel Network can be found in Supplementary Table 3.

Glomerular filtration rate and gout represented the disease pair with greatest number of shared neighbors (*n* = 10) in the novel disease network (Fig. 6). Five of these traits—lung adenocarcinoma, intelligence, squamous cell carcinoma, lung carcinoma and malaria—were connected not only to glomerular filtration rate and gout, but also to diabetes mellitus.

Our network also showed novel edges connecting rheumatoid arthritis (RA) to PAD and glomerular filtration rate (GFR). Previous studies have highlighted supporting evidence of the association between RA and GFR^52,53^ and RA and PAD^54–58^. Indeed, RA has pathological processes that also occurs in atherosclerosis, such as endothelial activation, inflammatory cell infiltration, neovascularization, and collagen degradation^59^. However, most studies investigating the association of rheumatoid arthritis with PAD are small and cross-sectional and future research is needed^54–58^.

These results (found in Supplementary Tables 4 and 5) highlight that genetics data revealed by MVP studies can help identify relationships among diseases that were not known before, indicating areas for future research related to disease mechanism, treatment, and prevention.

### Disease relationships driven by ancestry

It is well known that there exists some bias in genetic studies research, for which populations with European ancestry are over-represented in relation to other populations, such as Afro-American and Native American^39^. Therefore, we demonstrate these methods have the ability to characterize the landscape of disease-disease relationships driven by ancestry through distinguishing studies and GWAS results by separating European-only studies from all others.

**Figure 7:**
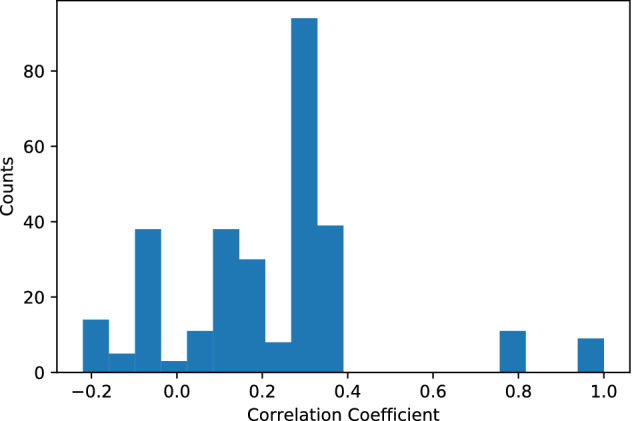
Community correlation between notes of different ancestry networks. Histogram of Pearson product-moment correlation coefficients for shared community members between the same trait in different ancestry networks. The count represents number of traits that have the given correlation coefficient.

We found that the community clusters profiled in the separate genetic networks are considerably different, with over 90% of nodes having less than a 0.4 correlation coefficient (Fig. 7). For example, we observed that hypertension, which had large difference in degree between the European and non-European networks (93 and 41, respectively), had an inverse correlation (− 0.22), demonstrating that it has a different profile of disease relationships in the two networks. In fact, blood pressure is a trait the has been found to be highly heritable, with substantial differences in blood pressure control rates between non-Hispanic white adults (55.7%) and non-Hispanic Blacks (48.5%)^60^. Therefore, GWAS studies with more diverse populations may allow the discovery of novel anti-hypertensive therapeutics by identifying new gene targets based on loci that have similar effect sizes across race/ethnic groups^47^.

Next, we explored the novel contributions that MVP has made by highlighting which edges in the European and non-European networks only occur in the presence of MVP publications. We found that, despite a large difference in size between the input data for these networks (155,760 and 47,749 SNP-trait associations, respectively), the graphs induced by the edges that only occur in MVP publications were relatively comparable in size (162 European edges vs 116 non-European edges). These results suggest that MVP has more heterogeneous population enabling investigation of both European and non-European based genetic relationships of diseases and their comorbidities.

## Discussion

In this study, we provide an overview of the relationship among phenotypes that share strong SNP-trait associations. We assembled a network of published genetic variants available through the GWAS Catalog repository to visualize novel connections and to investigate new insights gained through findings from numerous studies to-date. While recent studies^7,61–63^ have constructed disease networks through the use of known disease genes from sources such as the Online Mendelian Inheritance in Man (OMIM) and various GWAS databases, these networks are typically smaller in size and utilized as part of a further analysis such as exploring drug efficacy^61^, drug repurposing^63^ or revealing disease relationships based on expression levels^62^ or the interactome^7^. Our network reveals novel associations between diseases and provides a mechanistic approach to categorize diseases in different groups. Finally, we mapped the new disease associations that emerge only when we included variants from MVP studies contained within the GWAS Catalog. We believe that our results offer insights to better understand comorbidity patterns observed in patients and have the potential to reveal mechanistic links between diseases with further investigation. Additionally, the identification of diseases that share genetic similarities offers the opportunity to investigate possible drug-repurposing strategies for identification of new indications for existing drugs^61,63,64^.

We highlight that our approach relies only on genetic information, but diseases often manifest through multifaceted mechanisms including other clinical factors and shared environmental exposure^12,65^. Other approaches to evaluate disease relationships rely on connecting diseases that tend to co-occur in patients^5^ or for which patients usually show similar gene expression profiles^66–68^. Indeed, following the strategy from Klimek et al.^12^, a multi-layer network approach—where in each layer diseases are connected based on a different set of features (e.g., genetic variant or disease co-occurrence)—might distinguish driving forces in disease relationships that go beyond genetics information only^12^. We bring to attention that GWAS data may include non-causal variants that arise due to technical artifacts or other biological factors, such as a linkage disequilibrium. However, data availability on causal variants is very limited and specific to diseases of high clinical and research interest, resulting in studies highly affected by literature bias. We believe that big data analysis has the power to identify true biological signal even amidst high levels of noise. For example, previous network-medicine studies^4,7,61,63^ used GWAS-derived variants and were able to recover true disease-disease and disease drug relationships with high levels of predictive power. Machine learning-based models are also able to leverage on (non-causal or not) genetic variants to help reveal missing heritability and epistatic interactions on GWAS-based datasets^69^. Indeed, we also demonstrate that the proposed methodology identifies true biological signal by being able to recover clinically relevant disease relationships such as cancer and vitiligo^29,30^, Type II Diabetes and Alzheimer’s disease^32–37^, and Rheumatoid arthritis and PAD^54–58^. Furthermore, previous studies^63,70^ identified predictions that leverage GWAS-based variants and further validated observations with experimental and clinical data.

The results presented here aggregate the top hits from 3,985 studies found in the GWAS Catalog. Therefore, heterogeneity might exist in the definition of phenotypes across different studies. For example, the network contains 15 traits related to diabetes (Supplementary Table 6), containing broad definitions, such as diabetes mellitus, and more specific ones, such as type 2 diabetes nephropathy and diabetes mellitus type 2 associated cataract. However, we believe that, even in the presence of these variations, the general patterns observed here provide important insights for clinical practice. We also highlight that our study lays the foundations for future studies that could avoid these limitations by using GWAS data from well-phenotyped cohorts such as the MVP and UK Biobank. More specifically in the VA, there is a nation-wide effort to harmonize and catalog phenotypic mapping and algorithms where MVP is a major contributor. In addition, MVP has applied several advanced high-throughput phenotypic engines to develop complex phenotypes using large clinical database^71,72^. While MVP is a diverse cohort, it’s comprised of predominantly older men by design. However, due to the large size of the cohort, there are a substantial number in sub populations covering the rest of the general demographics. For instance, in a prior version of the MVP cohort (19.2), while women represented only 9.8% of the total cohort, there were still 64,658 individuals. Also, past MVP GWAS have found their results are able to be replicated^40,42,47,49,50,73,74^. Finally, our current study included only a part of the genetics data available in MVP and the GWAS Catalog by including studies added to the GWAS catalog before June 30^th^, 2020. Our results merit further investigation of more integrated network as the MVP and other major biobanks and cohorts continue to grow and produce next generation genetic discoveries.

## Methods

### Data

GWAS Catalog (version 1.0.2^19^) data was obtained and downloaded in July 2020 with a freeze on studies added on or before June 30, 2020, ensuring that the dataset used for analyses remained consistent and static. The GWAS catalog database included study information (i.e. lead author, study name, PubMedID, ancestry, study type), traits (mapped to ontology terms), and genetic variants that met the p-value threshold of 1 × 10^–5^. Additional criteria for inclusion in the catalog can be found elsewhere^19^.

The ontological system Experimental Factor Ontology (EFO)^75^ is used in the GWAS catalog to provide a level of consistency in the description of the traits. We used the EFO to map traits to their corresponding EFO categories (e.g. digestive system disorder, hematological measurements) and when multiple EFO terms could be mapped to the same trait, we assigned the trait to each possible term.

As our primary aim was to observe relatedness among diseases, we performed filtering steps to reduce the number of traits not directly related to diseases. We performed a regular expression search and removed all nodes with the keywords: "measurement" or "response to (medication/treatment)". This step removed 1,686 EFO terms or potential network nodes from consideration. It was important for us to retain as many disease nodes as possible and for this reason, we limited the number of keywords that would trigger trait elimination. We also removed from the network data 21 EFO terms that independently provided no meaning outside the context of their respective phenotype, such as "age at onset" and "age at diagnosis".

Traits related to the following EFO terms are determined not to be disease-related and therefore are not labeled in figures: other measurement, biological process, body measurement, lipid or lipoprotein measurement, response to drug, and hematological measurement.

Finally, for each study we obtained the trait and corresponding EFO term, the PubMedID, and the genetic variants. We used the PubMedIDs to differentiate studies belonging to research contributions of MVP.

### Network analysis

The network was created by using traits as nodes and by edges (or links) connected pairs of traits with shared variants. For each edge we calculate the normalized overlap (Jaccard Index) of variants between the pair of traits and applied the Fisher's exact test to assess the statistical significance of the overlap followed by Benjamini–Hochberg multiple testing correction. We performed community detection in the resulting network using the Louvain algorithm and the statistical significance of each community was evaluated following the strategy based on modularity and size, as proposed by Kojaku et al.^76^. The network analyses were performed with the Python packages ‘networkx’^77^ and ‘community’^21^ , statistical tests were performed with ‘Scipy’^78^ and ‘statsmodels’^79^ packages, and network visualization was performed with Cytoscape^80^.

Once the full disease related network was created from the GWAS catalog, we differentiated the networks for which there was no contribution from MVP studies from the network for which there was. We use the former to highlight the novel disease-disease relationships that emerge when MVP data is included.

To investigate the contribution of ancestry to our network we annotated the association data using a framework created by the GWAS Catalog team which contains ancestral categories for a given study^39^. Using this separate file provided by the GWAS Catalog to roll up more granular classifications into broader categories. For instance, ancestries labeled as “Sub-Saharan African” or “African unspecified” were collapsed into the category “African”. We then created indicator flags for each row in the catalog that highlights whether a study contained either European or non-European populations based on its study accession. These flags were not mutually exclusive. We then used the flags to replicate our network assembling pipeline and created two separate networks, European and non-European.

We then ask whether the diseases tend to have the same or different pattern of disease-disease connections in the European and non-European networks. We achieve this by representing each disease present in both networks (*n* = 300) with a vector of 0’s and 1’s, with 1’s indicating other conditions to which a disease is connected to in the same network and 0’s otherwise. By comparing the vectors of each disease in both networks, we were able to assess the extent to which their community profiles are similar or different.

Data used in this study are all publicly available from the GWAS Catalog which follows the General Data Protection Regulation (GDPR) as described on their website. The GWAS Catalog, a repository of summary statistics curated by the European Molecular Biology Laboratory, follows a time and release protocol where data is reviewed by a Data Access Committee before being released to the public. These research activities were approved by VA Central IRB #18-38.

## Supplementary Information

Supplementary Information.

## Data Availability

All data used was publicly available and downloaded from the GWAS catalog. More information can be found in the contents section of the Supplementary file.
